# iTRAQ-Based Quantitative Proteomic Analysis of Acinetobacter baumannii under Hypoxia and Normoxia Reveals the Role of OmpW as a Virulence Factor

**DOI:** 10.1128/spectrum.02328-21

**Published:** 2022-03-02

**Authors:** María Luisa Gil-Marqués, Jerónimo Pachón, Younes Smani

**Affiliations:** a Unit of Infectious Diseases, Microbiology and Preventive Medicine, Virgen del Rocío University Hospital, Seville, Spain; b Institute of Biomedicine of Seville, IBiS, University Hospital Virgen del Rocío/CSIC/University of Seville, Sevilla, Spain; c Department of Medicine, University of Seville, Sevilla, Spain; d CIBER de Enfermedades Infecciosas (CIBERINFEC), Instituto de Salud Carlos III, Madrid, Spain; e Department of Molecular Biology and Biochemical Engineering, Andalusian Center of Developmental Biology, CSIC, University of Pablo de Olavide, Seville, Spain; Griffith University

**Keywords:** hypoxia, *Acinetobacter baumannii*, iTRAQ, virulence factors, OmpW

## Abstract

Acinetobacter baumannii needs to adapt to hypoxia during infection. Understanding its proteome regulation during infection would allow us to determine new targets to develop novel treatments. iTRAQ proteomic analysis of A549 cell infection by the ATCC 17978 strain was performed. A total of 175 proteins were differentially expressed under hypoxia versus normoxia. We selected the hypoxia-downregulated protein OmpW to analyze its role as a virulence factor. The loss of OmpW decreased the adherence and invasion of A. baumannii in these host cells, without affecting its bacterial growth. Moreover, A549 cell viability with ΔOmpW infection was higher than that with the wild-type strain. ΔOmpW presented less biofilm formation. Finally, the minimum lethal dose required by the ΔOmpW mutant was higher than that of the wild-type strain in a murine peritoneal sepsis model, with lower bacterial loads in tissues and fluids. Therefore, OmpW seems to be a virulence factor necessary for A. baumannii pathogenesis.

**IMPORTANCE** Acinetobacter baumannii causes infections that are very difficult to treat due to the high rate of resistance to most and sometimes all of the antimicrobials used in the clinical setting. There is an important need to develop new strategies to combat A. baumannii infections. One alternative could be blocking specific bacterial virulence factors that this pathogen needs to infect cells. Pathogens modulate their protein expression as a function of the environment, and several studies have reported that hypoxia occurs in a wide range of infections. Therefore, it would be interesting to determine the proteome of A. baumannii under hypoxia in order to find new virulence factors, such as the outer membrane protein OmpW, as potential targets for the design of novel therapies.

## INTRODUCTION

Acinetobacter baumannii is an aerobic Gram-negative bacillus that has become an increasingly important human pathogen responsible for nosocomial infections (1). A. baumannii can develop resistance to all classes of antimicrobial agents used in the clinical setting (2, 3). Thus, it is important to identify new virulence factors to characterize the pathogenesis and determine new therapeutic targets (4).

Several studies show that bacteria modulate their gene expression and protein levels, and thus the expression of virulence factors, in different environments (5–7). It is known that hypoxia occurs during infection, with oxygen levels in the infection focus being <1%; this is due to increased oxygen consumption and decreased perfusion (8, 9). Moreover, we previously determined that hypoxia decreases the adherence and invasion of A. baumannii in human lung epithelial cells, without affecting bacterial growth (10). Thus, hypoxia might modify virulence factor expression during the course of infection.

Several genomic, transcriptomic, and proteomic analyses have identified different virulence factors that participate in the pathogenesis of A. baumannii, but we still know relatively few of them (1, 11–13). Techniques that use the relative quantification of the proteome, such as the isobaric tags for relative and absolute quantitation (iTRAQ) approach, have been used for characterization of bacterial proteomes, providing important information regarding virulence factors (7, 14–17). A. baumannii proteome analyses have also given new insights into the molecular mechanisms underlying its antibiotic resistance (18, 19), the growth-phase-dependent changes observed in its proteome (20), the molecular mechanisms involved in the response to desiccation and persistence (21), and proteins involved in biofilm formation (22). However, no study has investigated the influence of hypoxia on the A. baumannii proteome during infection.

The present study aimed to identify the A. baumannii proteins whose levels are regulated by hypoxia, to identify novel virulence factors. Thus, we compared protein levels in an infection model of human lung epithelial cells with A. baumannii ATCC 17978 under hypoxia (1% oxygen) and normoxia (21% oxygen) (10). We identified different upregulated and downregulated proteins under hypoxia. Further, we hypothesized that the outer membrane protein (OMP) most downregulated under hypoxia, OmpW (0.38-fold change), might be involved in the reduction in A. baumannii adherence to and invasion of host cells observed under hypoxia (10) and consequently in A. baumannii pathogenesis. We showed that an *ompW* deletion mutant presents less interaction with host cells *in vitro* and less virulence in an animal model, thus showing that OmpW is essential for *in vivo* infection by A. baumannii.

## RESULTS

### A. baumannii protein expression profile.

To identify proteins associated with an inducible virulence response in A. baumannii during infection, we searched for proteins that were differentially expressed under hypoxia versus normoxia (10). A total of 175 proteins were differentially expressed under hypoxia (51 proteins downregulated [10] and 124 proteins upregulated) (see Table S1 in the supplemental material). This accounts for 4% of the ATCC 17978 proteome.

**FIG 1:**
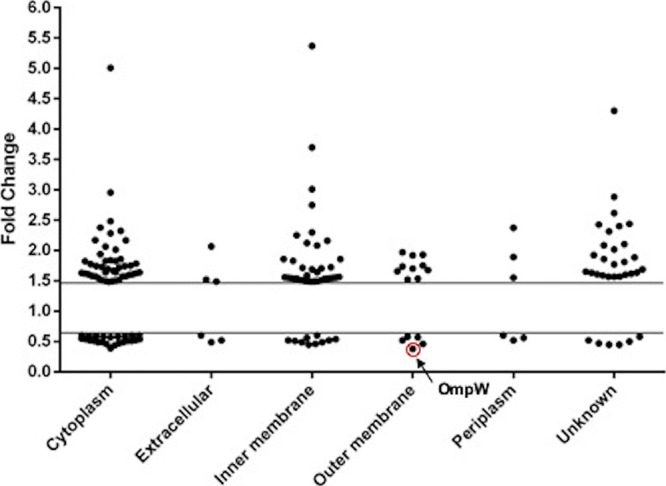
A. baumannii protein expression profile. Classification of downregulated (<0.6-fold change) and upregulated (>1.49-fold change) proteins under hypoxia were compared to those under normoxia according to their subcellular localization. Fifty-one downregulated proteins were localized in the cytoplasm (47.06%), extracellular medium (5.88%), inner membrane (19.61%), outer membrane (9.80%), and periplasm (5.88%). A total of 124 upregulated proteins were localized in the cytoplasm (36.29%), extracellular medium (2.42%), inner membrane (29.84%), outer membrane (8.06%), and periplasm (2.42%).

Most of the downregulated and upregulated proteins under hypoxia were localized in the cytoplasm (47.06 and 36.29%, respectively) and inner membrane (19.61 and 29.83%, respectively), although we also found proteins in the outer membrane, extracellular medium, and periplasm (Fig. 1). Downregulated proteins were mainly involved in metabolism and transport. Upregulated proteins were involved in metabolism, oxidative stress, DNA and RNA repair, transport, biogenesis of the fimbria, and transcriptional regulation. Furthermore, we observed that hypoxia modified the expression of OMPs. We found five downregulated OMPs, corresponding to OmpW (A1S_2325) and four ferric siderophores (A1S_3339, A1S_0474, A1S_0981, and A1S_1921) (10).

### Generation of OmpW mutants in A. baumannii.

The presence or absence of OmpW in the different strains was confirmed by PCR (see Fig. S1A) and quantitative reverse transcription-PCR (qRT-PCR) (see Fig. S1B). PCR showed that both wild-type strains (ATCC 17978 and ATCC 17978/p) and the complemented strain (ΔOmpW/pOmpW) yielded a band corresponding to the wild-type protein, while both mutants (ΔOmpW and ΔOmpW/p) yielded a band corresponding to *ompW* deletion (see Fig. S1A). We demonstrated by qRT-PCR that ATCC 17978 and ATCC 17978/p expressed similar levels of *ompW* RNA, while ΔOmpW/pOmpW showed overexpression of *ompW* due to the multicopy plasmid pUCp24 (see Fig. S1B). The ΔOmpW and ΔOmpW/p strains had no *ompW* expression.

### Effects of OmpW deletion on the growth of A. baumannii and on the bactericidal activity and bacterial cell adherence and invasion.

**FIG 2:**
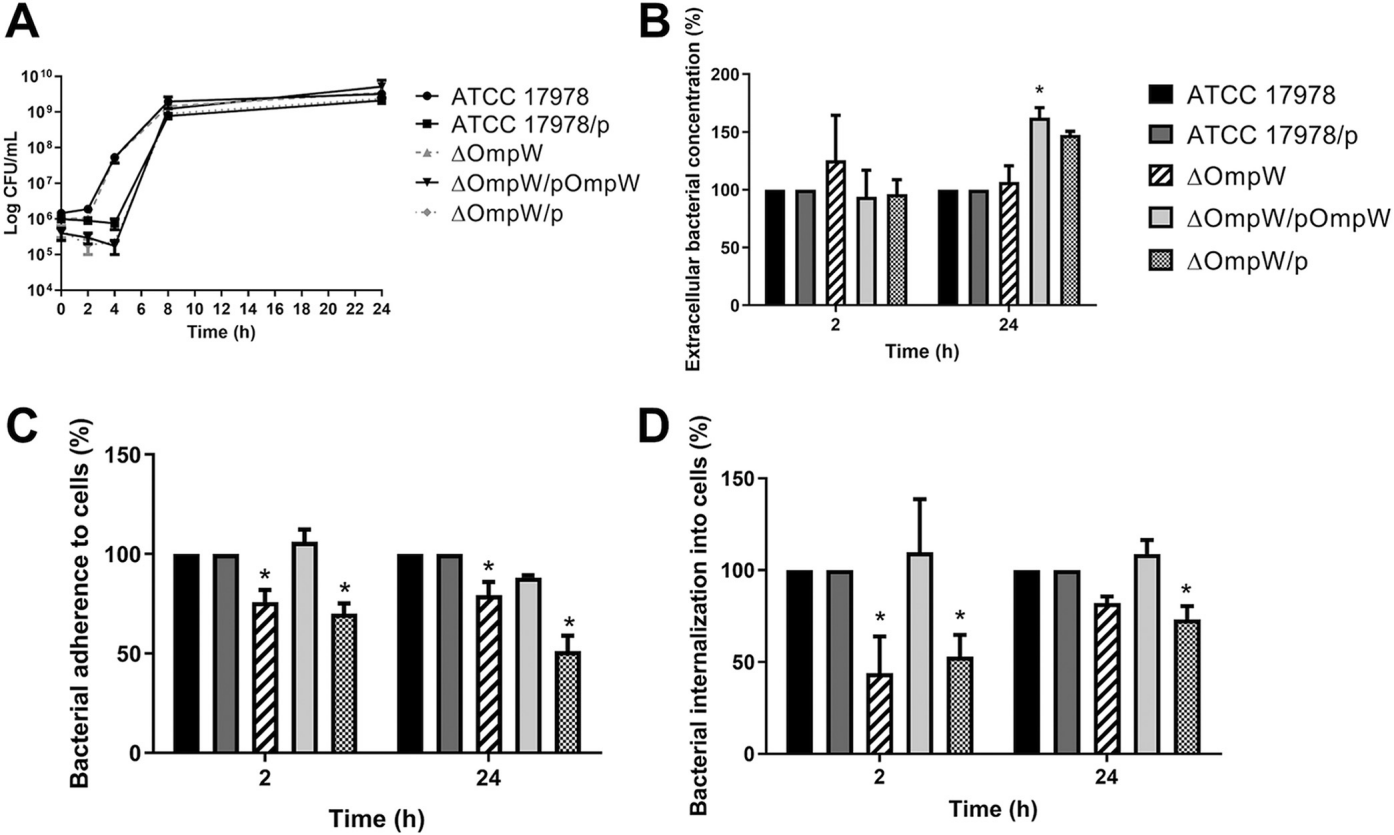
Effect of OmpW deletion on the growth of A. baumannii and on the bactericidal activity and bacterial cell adherence and invasion. (A) Growth curves for A. baumannii ATCC 17978, ATCC 17978/p, ΔOmpW, ΔOmpW/pOmpW, and ΔOmpW/p strains in MHB under normoxia. (B) Measurement of bacterial concentrations in the extracellular medium 2 and 24 h after A549 cell infection by A. baumannii ATCC 17978, ATCC 17978/p, ΔOmpW, ΔOmpW/pOmpW, and ΔOmpW/p strains under normoxia. (C) Measurement of bacterial adherence 2 and 24 h after A549 cell infection by A. baumannii ATCC 17978, ATCC 17978/p, ΔOmpW, ΔOmpW/pOmpW, and ΔOmpW/p strains under normoxia. (D) Measurement of bacterial invasion 2 and 24 h after A549 cell infection by A. baumannii ATCC 17978, ATCC 17978/p, ΔOmpW, ΔOmpW/pOmpW, and ΔOmpW/p strains under normoxia. *, *P < *0.05, versus the wild-type strain.

Growth curves under normoxia were analyzed to check ATCC 17978 and ΔOmpW growth rates. We showed that the growth of the two strains was indistinguishable. The complemented strain and strains harboring the empty pUCp24 plasmid had the same growth as the respective strains (Fig. 2A).

We wanted to determine whether the loss of OmpW affected the bactericidal activity of A549 cells against the mutant strain more than against the wild-type strain. Bacterial counts of ATCC 17978, ATCC 17978/p, ΔOmpW, ΔOmpW/pOmpW, and ΔOmpW/p strains in the extracellular medium of A549 cells were the same at 2 h postinfection (Fig. 2B). There was a significant increase in bacterial counts of ΔOmpW/pOmpW strain in the extracellular medium at 24 h postinfection (Fig. 2B). These data confirmed that the loss of OmpW did not affect the bactericidal activity of A549 cells.

Next, we determined whether the loss of OmpW affected the adherence of A. baumannii to these host cells. The adherence of the ΔOmpW strain was significantly lower than that of the wild-type strain at 2 and 24 h postinfection (75.98% versus 100% [*P < *0.05] and 79.29% versus 100% [*P < *0.05], respectively) (Fig. 2C), and the same was true for ΔOmpW/p (70.06% versus 100% [*P < *0.05] and 51.22% versus 100% [*P < *0.05], respectively) (Fig. 2C). Complementation of ΔOmpW restored the wild-type strain adherence.

Moreover, we determined whether the loss of OmpW affected the invasion of A. baumannii in A549 cells. Bacterial counts of the mutant strain inside A549 cells showed a decrease, compared to the wild-type strain, at 2 and 24 h postinfection (43.96% versus 100% [*P < *0.05] and 82% versus 100%, respectively) (Fig. 2D), and the same was true for ΔOmpW/p (52.90% versus 100% [*P < *0.05] and 73.23% versus 100% [*P < *0.05], respectively) (Fig. 2D). Complementation of ΔOmpW restored the wild-type strain invasion levels. These data indicated that the OmpW deletion diminished the adherence and invasion of A. baumannii in human lung epithelial cells.

### Role of OmpW in biofilm formation.

**FIG 3:**
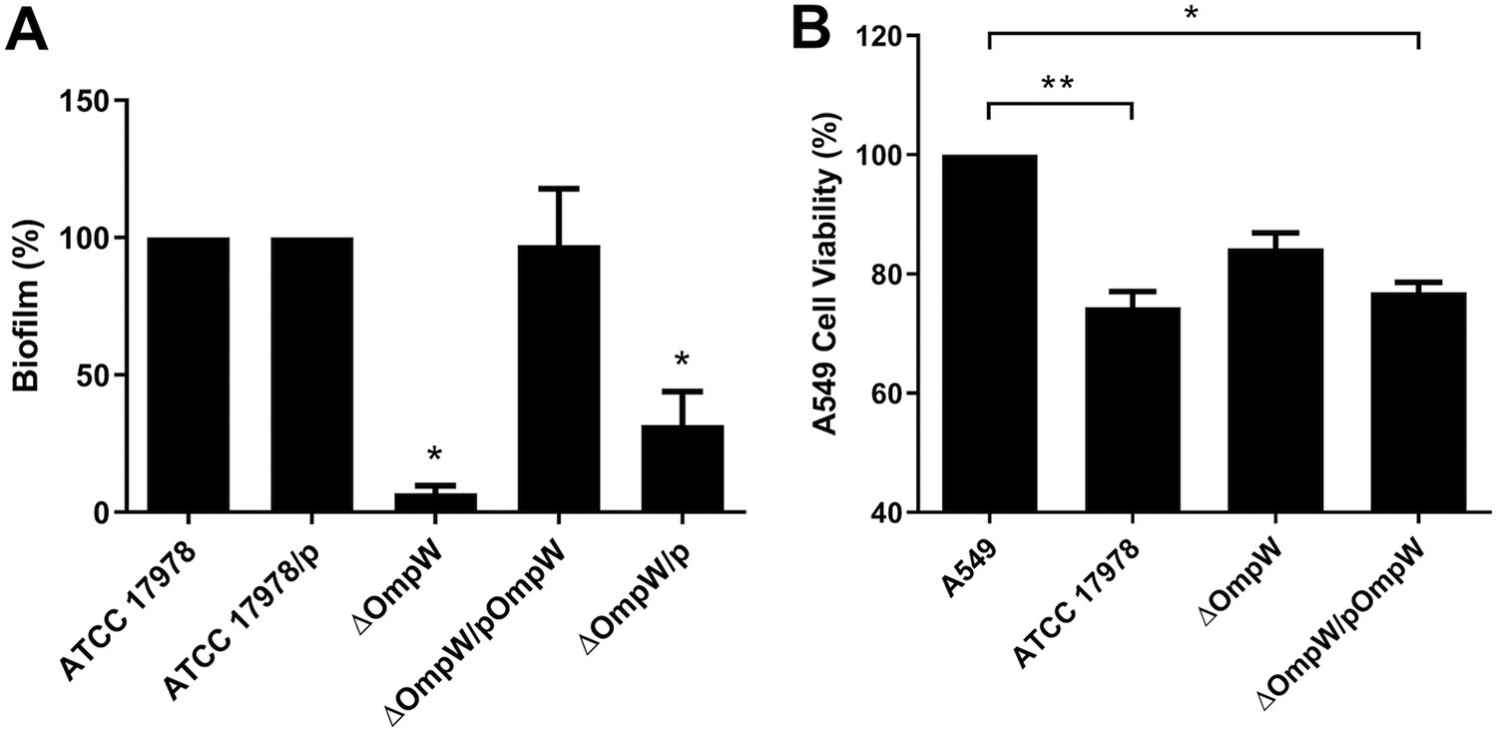
Role of OmpW in biofilm formation and in cellular viability with A. baumannii. (A) Effect of the loss of OmpW on biofilm production. Biofilm formation was determined for A. baumannii ATCC 17978, ATCC 17978/p, ΔOmpW, ΔOmpW/pOmpW, and ΔOmpW/p strains. (B) Viability of A549 cells after 24 h of infection with A. baumannii ATCC 17978, ΔOmpW, and ΔOmpW/pOmpW strains. *, *P < *0.05; **, *P < *0.01, versus the wild-type strain.

A biofilm assay was performed to determine whether the loss of OmpW affected the biofilm-forming potential of A. baumannii. The ATCC 17978 strain produced thick biofilm (Fig. 3A). However, the ΔOmpW strain demonstrated significantly less biofilm formation (6.89%), compared to the wild-type strain (100%), as did the ΔOmpW/p strain (31.64%) (*P < *0.05). Complementation of the ΔOmpW strain restored the biofilm production to the levels of the ATCC 17978/p strain (97.30%).

### Role of OmpW in cellular viability with A. baumannii.

We determined whether OmpW was involved in the cell death of A549 cells induced by A. baumannii. A cell survival assay showed that 24-h infection of A549 cells by the ATCC 17978 and ΔOmpW/pOmpW strains reduced cell viability to 74.41% and 76.94%, respectively. However, the ΔOmpW strain reduced the cell viability to 84.27% (Fig. 3B). These results showed that OmpW is involved in the cytotoxicity induced by A. baumannii.

### Effect of OmpW deletion on antibiotic susceptibility.

We wanted to determine how the deletion of OmpW in A. baumannii affected the susceptibility to some clinically relevant antibiotics. MIC values for the mutant strain were the same as those for the parental strain for all of the antibiotics tested (Table 1). These results indicated that deletion of OmpW did not affect the susceptibility of A. baumannii to these antibiotics.

**TABLE 1:** MIC values for the OmpW deletion mutant and its wild-type and complemented strains

Drug	MIC (mg/L) for strain:		
	ATCC 17978	ΔOmpW	ΔOmpW/pOmpW
Ceftazidime	8	8	8
Sulbactam	2	2	2
Imipenem	0.125	0.125	0.125
Amikacin	2	1	1
Azithromycin	2	2	2
Vancomycin	64	64	64
Rifampicin	1	2	1
Colistin	0.125	0.125	0.125
Ciprofloxacin	0.5	0.5	0.5
Tigecycline	0.5	0.5	0.5

### Role of OmpW in the virulence of A. baumannii in a murine peritoneal sepsis model.

**FIG 4:**
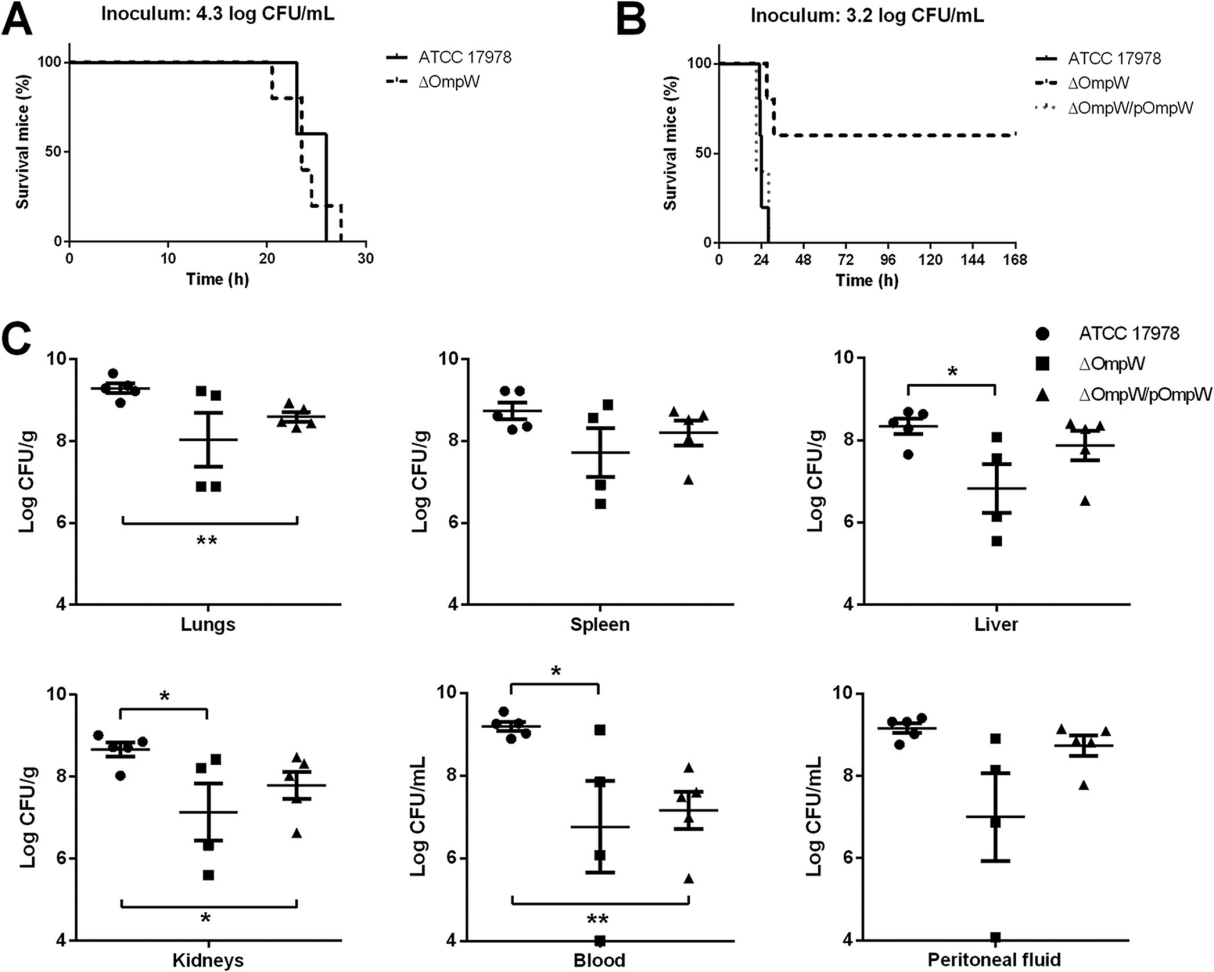
Role of OmpW in the virulence of A. baumannii in the murine peritoneal sepsis model. (A and B) Mortality curves analyzed by Kaplan-Meier survival analysis. *P < *0.01, versus the wild-type strain and for the mutant versus the complemented strain. (C) Bacterial loads in fluids and tissues in the murine peritoneal sepsis model with A. baumannii ATCC 17978, ΔOmpW, and ΔOmpW/pOmpW strains at 4.3 log CFU/mL. **, *P < *0.01; *, *P < *0.05, versus the wild-type strain.

To analyze the effect of OmpW on A. baumannii virulence, a murine model of peritoneal sepsis was used. The mortality rates were dependent on the concentration of bacteria in the inoculum for ATCC 17978, ΔOmpW, and ΔOmpW/pOmpW strains (Table 2). The 50% lethal dose (LD_50_) and 100% minimum lethal dose (MLD_100_) for the ΔOmpW strain were greater than those for the ATCC 17978 and ΔOmpW/pOmpW strains, i.e., 3.29 and 4.30 log CFU/mL versus 2.88 and 3.40 log CFU/mL, respectively, with ratios of 1.14 and 1.26, respectively. The 0% lethal dose (LD_0_) values for the ΔOmpW, ATCC 17978, and ΔOmpW/pOmpW strains were the same, i.e., 2.30 log CFU/mL (Table 2). Kaplan-Meier analysis showed differences between animal groups receiving the same inoculum of ΔOmpW and ATCC 17978 strains of 3.2 log CFU/mL (*P < *0.01). The group infected by the complemented strain had the same mortality rate as the wild-type strain (Fig. 4A and B).

**TABLE 2:** Seven-day mortality rates in the murine model of peritoneal sepsis with different inocula of Acinetobacter baumannii ATCC 17978 and its ΔOmpW mutant

Strain	Mortality rate (%) with inoculum of:		
	4.3 log CFU/mL	3.2 log CFU/mL	2.3 log CFU/mL
Wild-type ATCC 17978	100	100	0
ΔOmpW	100	40	0
ΔOmpW/pOmpW	100	100	0

Furthermore, we used the same murine model to compare the dissemination of the ATCC 17978, ΔOmpW, and ΔOmpW/pOmpW strains to different organs, inoculating mice intraperitoneally (i.p.) with each strain at 4.3 log CFU/mL. Collected tissues and fluids showed lower bacterial concentrations in mice infected by the ΔOmpW strain than in those infected by the ATCC 17978 strain (Fig. 4C). We found differences between the ΔOmpW and ATCC 17978 strains in liver (6.83 versus 8.33 log CFU/g [*P < *0.05]), kidney (7.14 versus 8.66 log CFU/g [*P < *0.05]), and peritoneal fluid (7.00 versus 9.19 log CFU/mL [*P < *0.05]). In the case of the ΔOmpW/pOmpW strain, bacterial loads in tissues and fluids were similar to those with the wild-type strain except for the blood and lung samples. These data indicated that OmpW plays an important role in the *in vivo* infective capacity of and mortality rates with A. baumannii.

## DISCUSSION

In this study, we report for the first time the role played by OmpW in the interaction of A. baumannii with human host cells and in the infectivity in a murine peritoneal sepsis model. We demonstrate that OmpW is downregulated in A. baumannii during infection of hypoxic human lung epithelial cells. Pathogenic bacteria need to adapt to diverse host environments during infection, such as hypoxic environments. Environmental signals trigger adaptive responses and induce the expression of virulence proteins (23). For this reason, it is important to know the proteome of A. baumannii during infection to design new treatments.

OmpW is an eight-stranded β-barrel porin that forms channels for the uptake of small hydrophobic molecules across the outer membrane (24). This protein is well conserved among A. baumannii laboratory and clinical strains (25) and in Gram-negative bacteria such Escherichia coli, Pseudomonas aeruginosa (OprG), Vibrio cholerae (26–28), Klebsiella pneumoniae, and Shigella flexneri (confirmed by BLASTp and ClustalW analyses) (see Fig. S2 in the supplemental material). It has been demonstrated to participate in bacterial adaptive responses to different environmental stresses (26, 28), although its function is not yet completely understood. OmpW participates in iron uptake in E. coli and A. baumannii (26, 29), in the response to oxidative stress in Salmonella enterica serovar Typhimurium (30), and in V. cholerae growth under hypersaline conditions (28). OmpW is upregulated under oxygen-limiting conditions or anaerobiosis in E. coli, Comamonas acidovorans, and P. aeruginosa (27, 31, 32). However, we showed that OmpW is downregulated in A. baumannii under hypoxia, suggesting the existence of different regulation pathways in E. coli (a facultative anaerobic pathogen) and A. baumannii (a strictly aerobe pathogen). Moreover, OmpW is involved in iron uptake (26, 29), and iron levels are low during bacterial infection as a defense mechanism, which might also explain why OmpW is downregulated during hypoxic infection. Finally, a promoter analysis shows that the *ompW* promoter has a Lrp binding box (see Table S2). Lrp (A1S_3307) is a transcriptional regulator that either activates or represses transcription, and it is upregulated (1.94-fold change) during hypoxic infection (see Table S1). Therefore, Lrp might downregulate OmpW during hypoxic infection.

Previously, we demonstrated that hypoxia affected the adherence and invasion of A. baumannii in human lung epithelial cells and murine macrophages (10). We found that OmpW is the most downregulated protein under hypoxia. Therefore, our study suggests that OmpW might be involved in the interaction of A. baumannii with host cells. A previous study of proteins that are involved in the attachment of Burkholderia cenocepacia to human lung epithelial cells also showed that OmpW was involved in bacterial adherence (33), which supports our hypothesis. Bacterial adhesion to and invasion into host cells are important steps to cause A. baumannii cytotoxicity (34). We observed that deletion of *ompW* reduced A. baumannii adherence/invasion into host cells, as well as its cytotoxicity. Of note, the ATCC 17978/pUCp24, ΔOmpW/pUCp24, and ΔOmpW/pUCp24-ompW strains have similar growth curves and lag times. However, we observed a decrease in the adherence and invasion of the ΔOmpW/pUCp24 strain, compared to the ATCC 17978/pUCp24 and ΔOmpW/pUCp24-ompW strains, which confirms that the lag time does not have any impact on the rest of the assays. These results are consistent with previous observations that, in the absence of OprG, which is homologous to OmpW in P. aeruginosa, this pathogen was significantly less cytotoxic against human bronchial epithelial cells (27).

It is noteworthy that biofilm plays an important role in bacterial pathogenesis, making A. baumannii more resistant to environmental stresses (23). In this study, we demonstrated that the deletion of *ompW* affects the biofilm formation by A. baumannii. In the same way, Ritter et al. identified OmpW and its homologue as inducers of biofilm by *Pseudoalteromonas* species and P. aeruginosa (35).

Furthermore, we demonstrated that *ompW* deletion in A. baumannii did not affect the susceptibility to multiple antibiotics belonging to different families. Previous studies reported a downregulation of OmpW in carbapenem- and colistin-resistant A. baumannii (36, 37). In contrast, two other studies demonstrated that OmpW was upregulated in carbapenem-resistant A. baumannii and was not associated with colistin resistance (20, 25). However, the question of how OmpW is relevant for antibiotic susceptibilities of A. baumannii has not been resolved.

OmpW is essential for A. baumannii to disseminate between organs and to cause the death of mice, as observed for other pathogens such as V. cholerae (38). Motley et al. reported an increase in OmpW expression during E. coli infection in a murine granulomatous pouch model (39), and OmpW has been shown to protect E. coli against host responses, conferring resistance to complement-mediated killing and phagocytosis (40, 41). All of those previous studies indicated that OmpW could be a potential drug target in Gram-negative bacteria to develop new treatments. Moreover, mice immunized with OmpW were less infected by B. cenocepacia, Burkholderia multivorans, and A. baumannii in a sepsis model (25, 33).

To summarize, we have identified that OmpW is differentially regulated under hypoxia in A. baumannii infection. OmpW seems to be a promising virulence factor that is involved in A. baumannii interaction with host cells *in vitro* and in its pathogenicity *in vivo*, but it does not affect the antimicrobial resistance profile. Nevertheless, a better understanding of the OmpW regulation is necessary to decipher its role in A. baumannii pathogenesis. Finally, inhibitors against this protein could be used as novel treatments in the future.

## MATERIALS AND METHODS

### Bacterial strain and growth curve assays.

The A. baumannii ATCC 17978 strain was used in the present study. Bacterial strains were grown overnight in Mueller-Hinton broth (MHB) (Sigma, Spain) under static conditions and then diluted 1:1,000 in a 40-mL culture in MHB. Bacterial strains were grown overnight in MHB at 160 rpm and 37°C for the eukaryotic cell culture experiments (human lung epithelial cell line A549), washed with phosphate-buffered saline (PBS) (Lonza, Spain), and suspended in Dulbecco’s modified Eagle’s medium (DMEM) (Gibco, Spain) before infection.

### A549 cell culture and bacterial infection.

A549 cells were grown in supplemented DMEM as described previously (10, 42). Briefly, in the case of hypoxia, cells were incubated in a hypoxic chamber (Coy Laboratories, USA) with 1% O_2_ at 37°C for 6 h before bacterial infection. Then, cells were seeded for 24 h, washed three times with PBS, incubated in DMEM, and infected at a multiplicity of infection (MOI) of 500.

### iTRAQ assay.

We analyzed the differential protein expression profiles between hypoxia (1% O_2_) and normoxia (21% O_2_) in A549 cells infected with ATCC 17978 by iTRAQ as described previously (10). Briefly, cells were collected in a lysis buffer composed of 1 M triethylammonium bicarbonate buffer (Sigma), 1:100 protease inhibitor cocktail (complete mini EDTA-free; Roche, Spain), 1:100 phosphatase inhibitor cocktail (PhosSTOP EASYpack; Roche), 0.05% SDS, and 0.002% benzonase (Novagen, USA) 2 h after bacterial infection. The supernatant was removed, and the protein concentration of the pellet was quantified (Qubit Life Technologies, USA). An iTRAQ 4-plex (reporters at 114 to 117; AB Sciex, Spain) was used. Samples were analyzed by nano-liquid chromatography (Nano-LC 100; Thermo Fisher Scientific, USA) and tandem mass spectrometry (MS/MS) (Q Exactive Plus Orbitrap; Thermo Electron, USA). Protein identification was performed using Proteome Discoverer v1.4, and MS/MS fragmentation patterns were identified by mapping against the UniProt database. The quantifiable proteins were those that were identified through >2 peptides with a confidence level of ≥95%, a *P* value of <0.05, and an error factor of <2 with every reference tag. Downregulated and upregulated proteins were considered when the fold change was <0.6 and >1.49, respectively. Subcellular localization was determined using the software PSORTb (http://www.psort.org/psortb).

### Mutant and complemented strain construction.

A stable, in-frame deletion mutant strain was constructed in ATCC 17978 by allelic exchange using the pMJG42 plasmid, which harbors the *sacB* gene for counterselection (43). For construction of the *ompW* deletion mutant (ΔOmpW), the 2,000 bp upstream and downstream of the open reading frame were amplified (see Table S3 in the supplemental material for primers). pMJG42 was digested with SpeI and NotI (New England Biolabs, USA). The Up insert was digested with SpeI and BamHI and the Down insert with BamHI and NotI. The plasmid and both inserts were ligated, and the construction was transformed into Escherichia coli DH5α λpir by electroporation before selection on LB agar plates with 5 mg/L tetracycline (Sigma). The construction was then transformed into E. coli MFD (44). The MFD donor strain harboring the respective pMJG42 gene (Up/Down) construct and the ATCC 17978 recipient strain were cultured overnight at 37°C. After 4 h of conjugation at 37°C, the cells were plated onto LB agar plates with 5 mg/L tetracycline. The colonies obtained were grown and plated on 10% sucrose plates. The OmpW deletion mutant was confirmed by PCR and sequencing using the primers OmpW-Out-F and OmpW-Out-R (see Table S3).

To complement the mutant, we followed the same protocol published by our research group (42). The open reading frame, 200 bp upstream, and 400 bp downstream were amplified with the primers NotI-OmpW-F and XbaI-OmpW-R. This DNA fragment was inserted into the pUCp24 plasmid and introduced by electroporation into the mutant strains before selection on LB agar plates with 10 mg/L gentamicin to obtain the complemented strain (ΔOmpW/pOmpW). Primers Seq-insert-pUCp24-F and Seq-insert-pUCp24-R were used to sequence the cloned gene. The wild-type strain and the deletion mutant strain were also transformed with the empty pUCp24 plasmid for use as a control (ATCC 17978/p and ΔOmpW/p, respectively).

### qRT-PCR assay.

Bacterial RNA was purified using the RNeasy minikit (Qiagen, Germany), and RT was carried out using the QuantiTect RT kit (Qiagen). We selected the primers 5′-AGCGGGTGGAGATATTCCTT-3′ (forward) and 5′-CACGCCAGCTCCGATATAAG-3′ (reverse) to amplify OmpW. We used the gene *rpoD* (*A1S_2706*) as a housekeeping gene (forward, 5′-CATGCGTGAAATGGGTACAG-3′; reverse, 5′-TTACTGGCCAAATGCTGTTG-3′). The qRT-PCR was carried out with SYBR Premix *Ex Taq* (TaKaRa, Japan) using a MxPro 3005p system (Stratagene, USA). Three technical replicates for each sample were included. The amplification conditions were as follows: 95°C for 30 s, followed by 35 cycles of 95°C for 10 s, 56°C for 25 s, and 72°C for 25 s. Relative quantification of gene expression was performed with the comparative threshold cycle (*C_T_*) method (Applied Biosystems guide).

### OmpW protein alignments and promoter analysis.

OmpW alignments between different bacterial pathogens were performed using BLASTp (https://blast.ncbi.nlm.nih.gov/Blast.cgi?PAGE=Proteins) and represented with Clustal (https://www.ebi.ac.uk/Tools/msa/clustalo) and Jalview (https://www.jalview.org). The promoter of *ompW* was analyzed with BPROM (http://www.softberry.com/berry.phtml?topic=bprom&group=programs&subgroup=gfindb) to find consensus boxes for transcription factors in its promoter region.

### Bactericidal activity and bacterial cell adherence and invasion.

After A549 cell infection with bacterial strains under normoxic conditions, extracellular medium was collected to determine bacterial concentrations at 2 and 24 h after bacterial infection (10).

Adherence and invasion assays were performed in triplicate as reported previously (10). Briefly, for adherence assays, cells were infected (MOI of 500), washed with PBS, and lysed with Triton X-100 (Sigma). The invasion assays were carried out in the same way as the adherence assay and included a treatment with 256 μg/mL tetracycline (Sigma) before lysis with Triton X-100.

### Cellular viability assay.

A549 cells were infected with an MOI of 500 for 24 h, as described previously (45). The 3-(4,5-dimethylthiazol-2-yl)-2,5-diphenyltetrazolium bromide (MTT) assay (Sigma) was performed in triplicate to determine the cell viability.

### Biofilm assay.

The biofilm assay was based on a previously described protocol (46). Briefly, strains were cultured overnight at 160 rpm and 37°C and diluted to 1 × 10^5^ CFU/mL. Two hundred microliters of the suspension was added to a 96-well plate and grown overnight at 37°C. Every well was washed and filled with 0.4% crystal violet (Sigma) and then incubated for 10 min. Each well was then washed and filled with 96% ethanol. Biofilm formation was determined after 15 min by measuring the optical density at 580 nm (Asys UVM 340 microplate reader; Biochrom, USA). This assay was performed in triplicate.

### Antibiotic susceptibility testing.

MICs were determined by broth microdilution according to CLSI guidelines (47). E. coli ATCC 25922 and Staphylococcus aureus ATCC 29213 strains were used as quality controls.

### Murine model of peritoneal sepsis. (i) Animals.

Immunocompetent C57BL/6 female mice (20 g) (Production and Experimentation Animal Center, University of Seville, Seville, Spain) were used. This study was performed following directive 2010/63/EU on the protection of animals used for scientific research. Experiments were approved by the Committee on the Ethics of Animal Experiments of the University Hospital of Virgen del Rocío (Seville, Spain) (approval number 20-05-14-84). All procedures were performed under sodium thiopental (B. Braun Medical S.A., Spain) anesthesia, and all efforts were made to minimize suffering.

### (ii) Experimental model.

A murine peritoneal sepsis model with A. baumannii was established by i.p. inoculation of bacteria (10). Animals were inoculated with 0.5 mL of the bacterial suspension, mixed 1:1 with a saline solution of porcine mucin (Sigma) at 10% (wt/vol). Groups of 5 mice for each strain were inoculated with different bacterial concentrations, and the survival of the mice was followed for 7 days. LD_0_ and MLD_100_ values were determined. LD_50_ values were calculated using the probit method (48). To measure *in vivo* dissemination, groups of 5 mice were inoculated i.p. with 4.3 log CFU/mL. Mice were sacrificed by i.p. injection of 200 μL sodium thiopental after 12 h of infection. Spleens, lungs, kidneys, and livers were aseptically extracted and homogenized in 2 mL of saline solution using a Stomacher 80 homogenizer (Tekmar Co., USA). Bacterial loads in these tissues (log_10_ CFU per gram) and bacterial concentrations in blood and peritoneal fluid (log_10_ CFU per milliliter) were quantified.

### Statistical analysis.

Data are presented as means ± standard errors of the mean (SEMs). Significant differences were determined using the Kruskal-Wallis test with Dunn *post hoc* testing (IBM SPSS Statistics 22 software). For peritoneal sepsis survival, a Kaplan-Meier test was performed to determine mortality rate differences. Statistical significance was considered when *P* values were <0.05.

### Data availability.

The data that support the findings of this study are available from the corresponding author upon reasonable request. The proteomic data were deposited in the PeptideAtlas database with the identifier PASS01733.
