# Supplementary material for: iTRAQ-Based Quantitative Proteomic Analysis of Acinetobacter baumannii under Hypoxia and Normoxia Reveals the Role of OmpW as a Virulence Factor
Source: Microbiol Spectr. 2022 Mar 2;10(2):e02328-21. doi: 10.1128/spectrum.02328-21 (PMC8941935; doi:10.1128/spectrum.02328-21)
Supplement: SUPPLEMENTAL FILE 1 — Supplemental material. Download SPECTRUM02328-21_Supp_1_seq4.pdf, PDF file, 0.8 MB [file spectrum02328-21_supp_1_seq4.pdf]

Supplemental Material

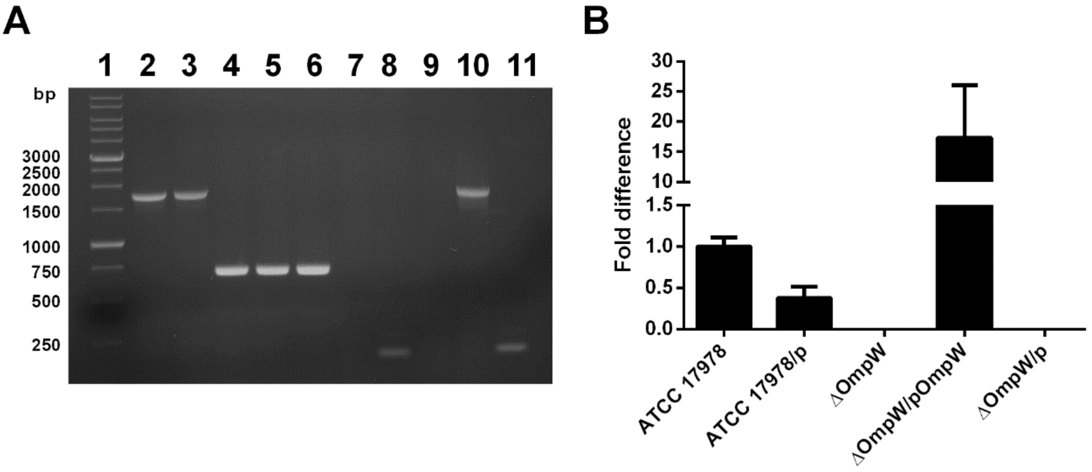

Figure S1. Generation of OmpW mutant in *A. baumannii.* **A)** PCR of *A. baumannii* ATCC 17978 (lanes 2 and 7), ATCC 17978/p (lanes 3 and 8), ΔOmpW (lanes 4 and 9), ΔOmpW/pOmpW (lanes 5 and 10), and ΔOmpW/p (lanes 6 and 11) strains. The lanes 2-6 correspond to the PCR using the primers OmpW Out F and OmpW Out R. The lanes 7-11 correspond to the PCR using the primers Seq.insert.pUCp24.Forward and Seq.insert.pUCp24.Reverse. Band sizes: 1797 bp (lanes 2 and 3), 696 bp (lanes 4-6), 128 bp (lanes 8 and 11), and 1863 bp (lane 10). **B)** qRT-PCR of *A. baumannii* ATCC 17978, ATCC 17978/p, ΔOmpW, ΔOmpW/pOmpW, and ΔOmpW/p strains to determine *ompW* RNA levels.

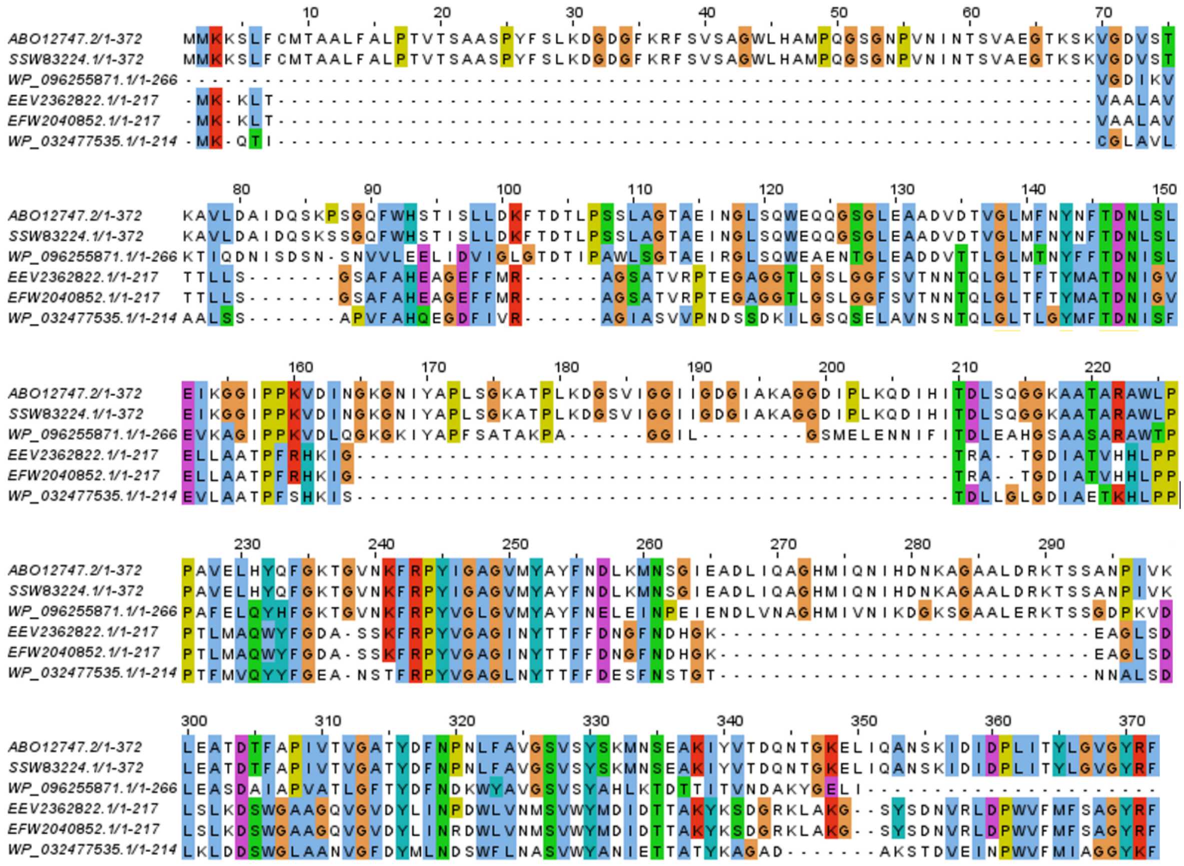

Figure S2. Clustal alignment of OmpW homologs. The OmpW protein from *A. baumannii* ATCC 17978 (ABO12747.2) was aligned with *K. pneumoniae* OmpW (SSW83224.1, 99.73% of identity with ABO12747.2), *P. aeruginosa* (WP_096255871.1, 56.03% of identity), *E. coli* (EEV2362822.1, 27.04% of identity with ABO12747.2), *S. flexneri* (EFW2040852.1, 26.42% of identity), and *V. cholerae* (WP_032477535.1, 25.52% of identity).

Table S1. Identification of the *A. baumannii* ATCC 17978 upregulated proteins under hypoxia condition (1% O_2).

Accession	Description	Fold Change
A3M7U7	23-dihydroxybenzoate-AMP ligase OS=*Acinetobacter baumannii* (strain ATCC 17978 / CIP 53.77 / LMG 1025 / NCDC KC755 / 5377) GN=A1S_2574 PE=4 SV=2 - [A3M7U7_ACIBT]	1.49
A3M1P4	Uncharacterized protein OS=*Acinetobacter baumannii* (strain ATCC 17978 / CIP 53.77 / LMG 1025 / NCDC KC755 / 5377) GN=A1S_0379 PE=4 SV=2 - [A3M1P4_ACIBT]	1.49
A3M6H4	Putative exported protein OS=*Acinetobacter baumannii* (strain ATCC 17978 / CIP 53.77 / LMG 1025 / NCDC KC755 / 5377) GN=A1S_2091 PE=4 SV=2 - [A3M6H4_ACIBT]	1.49
A3M4I6	Putative amino acid efflux transmembrane protein OS=*Acinetobacter baumannii* (strain ATCC 17978 / CIP 53.77 / LMG 1025 / NCDC KC755 / 5377) GN=A1S_1402 PE=4 SV=2 - [A3M4I6_ACIBT]	1.49
A3M4F4	Oxidoreductase OS=*Acinetobacter baumannii* (strain ATCC 17978 / CIP 53.77 / LMG 1025 / NCDC KC755 / 5377) GN=A1S_1370 PE=4 SV=1 - [A3M4F4_ACIBT]	1.49
A3M7E7	50S ribosomal protein L31 OS=*Acinetobacter baumannii* (strain ATCC 17978 / CIP 53.77 / LMG 1025 / NCDC KC755 / 5377) GN=rpmE PE=3 SV=1 - [RL31_ACIBT]	1.49
A3M5N6	Uncharacterized protein OS=*Acinetobacter baumannii* (strain ATCC 17978 / CIP 53.77 / LMG 1025 / NCDC KC755 / 5377) GN=A1S_1803 PE=4 SV=2 - [A3M5N6_ACIBT]	1.49
A3M194	Uncharacterized protein OS=*Acinetobacter baumannii* (strain ATCC 17978 / CIP 53.77 / LMG 1025 / NCDC KC755 / 5377) GN=A1S_0212 PE=4 SV=2 - [A3M194_ACIBT]	1.50
A3M6J0	Putative glutamine amidotransferase OS=*Acinetobacter baumannii* (strain ATCC 17978 / CIP 53.77 / LMG 1025 / NCDC KC755 / 5377) GN=A1S_2107 PE=4 SV=2 - [A3M6J0_ACIBT]	1.50
A3M700	RNA-splicing ligase RtcB OS=*Acinetobacter baumannii* (strain ATCC 17978 / CIP 53.77 / LMG 1025 / NCDC KC755 / 5377) GN=rtcB PE=3 SV=2 - [A3M700_ACIBT]	1.50
A3M6X5	Uncharacterized protein OS=*Acinetobacter baumannii* (strain ATCC 17978 / CIP 53.77 / LMG 1025 / NCDC KC755 / 5377) GN=A1S_2246 PE=4 SV=2 - [A3M6X5_ACIBT]	1.50
A3M343	Putative D-amino acid oxidase OS=*Acinetobacter baumannii* (strain ATCC 17978 / CIP 53.77 / LMG 1025 / NCDC KC755 / 5377) GN=A1S_0905 PE=4 SV=2 - [A3M343_ACIBT]	1.51

A3M6Q0	Cytochrome o ubiquinol oxidase subunit I OS=*Acinetobacter baumannii* (strain ATCC 17978 / CIP 53.77 / LMG 1025 / NCDC KC755 / 5377) GN=A1S_2167 PE=3 SV=2 - [A3M6Q0_ACIBT]	1.51
A3M5Z0	Putative peroxidase OS=*Acinetobacter baumannii* (strain ATCC 17978 / CIP 53.77 / LMG 1025 / NCDC KC755 / 5377) GN=A1S_1907 PE=4 SV=2 - [A3M5Z0_ACIBT]	1.51
A3M4R7	Methionine import ATP-binding protein MetN OS=*Acinetobacter baumannii* (strain ATCC 17978 / CIP 53.77 / LMG 1025 / NCDC KC755 / 5377) GN=metN PE=3 SV=2 - [A3M4R7_ACIBT]	1.51
A3M9I0	Fimbrial protein OS=*Acinetobacter baumannii* (strain ATCC 17978 / CIP 53.77 / LMG 1025 / NCDC KC755 / 5377) GN=A1S_3177 PE=3 SV=1 - [A3M9I0_ACIBT]	1.52
A3M185	Putative signal peptide OS=*Acinetobacter baumannii* (strain ATCC 17978 / CIP 53.77 / LMG 1025 / NCDC KC755 / 5377) GN=A1S_0202 PE=4 SV=2 - [A3M185_ACIBT]	1.52
A3M7C9	Uncharacterized protein OS=*Acinetobacter baumannii* (strain ATCC 17978 / CIP 53.77 / LMG 1025 / NCDC KC755 / 5377) GN=A1S_2404 PE=4 SV=2 - [A3M7C9_ACIBT]	1.53
A3M8V7	Uncharacterized protein OS=*Acinetobacter baumannii* (strain ATCC 17978 / CIP 53.77 / LMG 1025 / NCDC KC755 / 5377) GN=A1S_2946 PE=4 SV=2 - [A3M8V7_ACIBT]	1.54
A3M7Y3	Competence factor involved in DNA uptake OS=*Acinetobacter baumannii* (strain ATCC 17978 / CIP 53.77 / LMG 1025 / NCDC KC755 / 5377) GN=A1S_2610 PE=4 SV=2 - [A3M7Y3_ACIBT]	1.54
A7FBB0	Uncharacterized protein OS=*Acinetobacter baumannii* (strain ATCC 17978 / CIP 53.77 / LMG 1025 / NCDC KC755 / 5377) GN=A1S_3673 PE=4 SV=1 - [A7FBB0_ACIBT]	1.54
A3M308	Putative metal-dependent hydrolase OS=*Acinetobacter baumannii* (strain ATCC 17978 / CIP 53.77 / LMG 1025 / NCDC KC755 / 5377) GN=A1S_0870 PE=4 SV=2 - [A3M308_ACIBT]	1.54
A3M4Z2	Type 4 fimbrial biogenesis protein OS=*Acinetobacter baumannii* (strain ATCC 17978 / CIP 53.77 / LMG 1025 / NCDC KC755 / 5377) GN=A1S_1559 PE=4 SV=2 - [A3M4Z2_ACIBT]	1.54
A3M2S3	Putative threonine efflux protein (RhtC) OS=*Acinetobacter baumannii* (strain ATCC 17978 / CIP 53.77 / LMG 1025 / NCDC KC755 / 5377) GN=A1S_0777 PE=4 SV=2 - [A3M2S3_ACIBT]	1.54
A3M1A1	Transcriptional repressor NrdR OS=*Acinetobacter baumannii* (strain ATCC 17978 / CIP 53.77 / LMG 1025 / NCDC KC755 / 5377) GN=nrdR PE=3 SV=2 - [NRDR_ACIBT]	1.55
A3M5P7	Putative transporter OS=*Acinetobacter baumannii* (strain ATCC 17978 / CIP 53.77 / LMG 1025 / NCDC KC755 / 5377) GN=A1S_1814 PE=4 SV=2 - [A3M5P7_ACIBT]	1.55

A3M731	ABC Lysine-arginine-ornithine transporter periplasmic ligand binding protein OS=*Acinetobacter baumannii* (strain ATCC 17978 / CIP 53.77 / LMG 1025 / NCDC KC755 / 5377) GN=A1S_2302 PE=3 SV=2 - [A3M731_ACIBT]	1.55
A3M1E7	Putative membrane protein OS=*Acinetobacter baumannii* (strain ATCC 17978 / CIP 53.77 / LMG 1025 / NCDC KC755 / 5377) GN=A1S_0266 PE=4 SV=2 - [A3M1E7_ACIBT]	1.55
A3M3A6	Putative phthalate transporter OS=*Acinetobacter baumannii* (strain ATCC 17978 / CIP 53.77 / LMG 1025 / NCDC KC755 / 5377) GN=A1S_0968 PE=4 SV=2 - [A3M3A6_ACIBT]	1.56
A3M175	Putative transport protein (MFS superfamily) OS=*Acinetobacter baumannii* (strain ATCC 17978 / CIP 53.77 / LMG 1025 / NCDC KC755 / 5377) GN=A1S_0188 PE=4 SV=2 - [A3M175_ACIBT]	1.56
A3M426	Putative ABC family drug transporter OS=*Acinetobacter baumannii* (strain ATCC 17978 / CIP 53.77 / LMG 1025 / NCDC KC755 / 5377) GN=A1S_1242 PE=4 SV=2 - [A3M426_ACIBT]	1.56
A3M1I3	Putative acyl-CoA thioesterase II OS=*Acinetobacter baumannii* (strain ATCC 17978 / CIP 53.77 / LMG 1025 / NCDC KC755 / 5377) GN=A1S_0311 PE=4 SV=2 - [A3M1I3_ACIBT]	1.57
A3M3E0	Uncharacterized protein OS=*Acinetobacter baumannii* (strain ATCC 17978 / CIP 53.77 / LMG 1025 / NCDC KC755 / 5377) GN=A1S_1002 PE=4 SV=1 - [A3M3E0_ACIBT]	1.57
A3M2Y3	Putative flavodoxin or tryptophan repressor binding protein OS=*Acinetobacter baumannii* (strain ATCC 17978 / CIP 53.77 / LMG 1025 / NCDC KC755 / 5377) GN=A1S_0843 PE=4 SV=2 - [A3M2Y3_ACIBT]	1.57
A3M1X9	Non-canonical purine NTP pyrophosphatase OS=*Acinetobacter baumannii* (strain ATCC 17978 / CIP 53.77 / LMG 1025 / NCDC KC755 / 5377) GN=A1S_0468 PE=3 SV=2 - [NTPA_ACIBT]	1.57
A3M2V2	Malonyl-[acyl-carrier protein] O-methyltransferase OS=*Acinetobacter baumannii* (strain ATCC 17978 / CIP 53.77 / LMG 1025 / NCDC KC755 / 5377) GN=bioC PE=3 SV=2 - [A3M2V2_ACIBT]	1.57
A3M3N7	Probable 5-dehydro-4-deoxyglucarate dehydratase OS=*Acinetobacter baumannii* (strain ATCC 17978 / CIP 53.77 / LMG 1025 / NCDC KC755 / 5377) GN=A1S_1101 PE=3 SV=2 - [KDGD_ACIBT]	1.58
A3M9S0	Putative methyltransferase OS=*Acinetobacter baumannii* (strain ATCC 17978 / CIP 53.77 / LMG 1025 / NCDC KC755 / 5377) GN=A1S_3275 PE=4 SV=2 - [A3M9S0_ACIBT]	1.58
A3M169	Uncharacterized protein OS=*Acinetobacter baumannii* (strain ATCC 17978 / CIP 53.77 / LMG 1025 / NCDC KC755 / 5377) GN=A1S_0182 PE=4 SV=2 - [A3M169_ACIBT]	1.59
A3M4R9	D-methionine transport protein OS=*Acinetobacter baumannii* (strain ATCC 17978 / CIP 53.77 / LMG 1025 / NCDC KC755 / 5377) GN=A1S_1485 PE=4 SV=2 - [A3M4R9_ACIBT]	1.59

A3M590	Putative siderophore biosynthesis protein putative acetyltransferase OS=*Acinetobacter baumannii* (strain ATCC 17978 / CIP 53.77 / LMG 1025 / NCDC KC755 / 5377) GN=A1S_1657 PE=4 SV=2 - [A3M590_ACIBT]	1.60
A3M4M0	Putative acyl-CoA dehydrogenase OS=*Acinetobacter baumannii* (strain ATCC 17978 / CIP 53.77 / LMG 1025 / NCDC KC755 / 5377) GN=A1S_1436 PE=4 SV=1 - [A3M4M0_ACIBT]	1.60
A3M998	ATP-dependent DNA helicase RecG OS=*Acinetobacter baumannii* (strain ATCC 17978 / CIP 53.77 / LMG 1025 / NCDC KC755 / 5377) GN=recG PE=3 SV=2 - [A3M998_ACIBT]	1.61
A3M5R5	Oxidoreductase FMN-binding OS=*Acinetobacter baumannii* (strain ATCC 17978 / CIP 53.77 / LMG 1025 / NCDC KC755 / 5377) GN=A1S_1832 PE=4 SV=2 - [A3M5R5_ACIBT]	1.61
A3M8U2	Uncharacterized protein OS=*Acinetobacter baumannii* (strain ATCC 17978 / CIP 53.77 / LMG 1025 / NCDC KC755 / 5377) GN=A1S_2931 PE=4 SV=2 - [A3M8U2_ACIBT]	1.61
A3M3L8	Uncharacterized protein OS=*Acinetobacter baumannii* (strain ATCC 17978 / CIP 53.77 / LMG 1025 / NCDC KC755 / 5377) GN=A1S_1082 PE=4 SV=1 - [A3M3L8_ACIBT]	1.62
A3M684	Molybdenum cofactor guanylyltransferase OS=*Acinetobacter baumannii* (strain ATCC 17978 / CIP 53.77 / LMG 1025 / NCDC KC755 / 5377) GN=mobA PE=3 SV=2 - [MOBA_ACIBT]	1.62
A3M445	Putative 3-hydroxyacyl-CoA dehydrogenase OS=*Acinetobacter baumannii* (strain ATCC 17978 / CIP 53.77 / LMG 1025 / NCDC KC755 / 5377) GN=A1S_1261 PE=4 SV=2 - [A3M445_ACIBT]	1.63
A3M5D4	Dihydrolipoamide acetyltransferase OS=*Acinetobacter baumannii* (strain ATCC 17978 / CIP 53.77 / LMG 1025 / NCDC KC755 / 5377) GN=A1S_1701 PE=3 SV=1 - [A3M5D4_ACIBT]	1.63
A3M5K3	Uncharacterized protein OS=*Acinetobacter baumannii* (strain ATCC 17978 / CIP 53.77 / LMG 1025 / NCDC KC755 / 5377) GN=A1S_1770 PE=4 SV=2 - [A3M5K3_ACIBT]	1.63
A3M3E7	Putative lipoprotein OS=*Acinetobacter baumannii* (strain ATCC 17978 / CIP 53.77 / LMG 1025 / NCDC KC755 / 5377) GN=A1S_1009 PE=4 SV=2 - [A3M3E7_ACIBT]	1.64
A3M5M6	Transcriptional regulator LysR family OS=*Acinetobacter baumannii* (strain ATCC 17978 / CIP 53.77 / LMG 1025 / NCDC KC755 / 5377) GN=A1S_1793 PE=4 SV=2 - [A3M5M6_ACIBT]	1.64
A3M2N6	Putative phage related protein OS=*Acinetobacter baumannii* (strain ATCC 17978 / CIP 53.77 / LMG 1025 / NCDC KC755 / 5377) GN=A1S_0740 PE=4 SV=2 - [A3M2N6_ACIBT]	1.64
A3M255	Putative membrane protein OS=*Acinetobacter baumannii* (strain ATCC 17978 / CIP 53.77 / LMG 1025 / NCDC KC755 / 5377) GN=A1S_0546 PE=4 SV=2 - [A3M255_ACIBT]	1.64

A3M2G3	Transposase OS=*Acinetobacter baumannii* (strain ATCC 17978 / CIP 53.77 / LMG 1025 / NCDC KC755 / 5377) GN=A1S_0658 PE=4 SV=2 - [A3M2G3_ACIBT]	1.64
A7FBX4	Uncharacterized protein OS=*Acinetobacter baumannii* (strain ATCC 17978 / CIP 53.77 / LMG 1025 / NCDC KC755 / 5377) GN=A1S_3887 PE=4 SV=1 - [A7FBX4_ACIBT]	1.65
A3M9L6	Putative RND family drug transporter OS=*Acinetobacter baumannii* (strain ATCC 17978 / CIP 53.77 / LMG 1025 / NCDC KC755 / 5377) GN=A1S_3219 PE=4 SV=2 - [A3M9L6_ACIBT]	1.66
A3M5U7	Acyl-CoA dehydrogenase-like protein OS=*Acinetobacter baumannii* (strain ATCC 17978 / CIP 53.77 / LMG 1025 / NCDC KC755 / 5377) GN=A1S_1864 PE=4 SV=2 - [A3M5U7_ACIBT]	1.66
A3M5A0	Putative ferric hydroxamate siderophore receptor OS=*Acinetobacter baumannii* (strain ATCC 17978 / CIP 53.77 / LMG 1025 / NCDC KC755 / 5377) GN=A1S_1667 PE=3 SV=2 - [A3M5A0_ACIBT]	1.68
A3M6X9	Putative membrane protein OS=*Acinetobacter baumannii* (strain ATCC 17978 / CIP 53.77 / LMG 1025 / NCDC KC755 / 5377) GN=A1S_2250 PE=4 SV=2 - [A3M6X9_ACIBT]	1.69
A3M8R5	Uncharacterized protein OS=*Acinetobacter baumannii* (strain ATCC 17978 / CIP 53.77 / LMG 1025 / NCDC KC755 / 5377) GN=A1S_2903 PE=4 SV=2 - [A3M8R5_ACIBT]	1.69
A3M8Y0	Putative vanillate O-demethylase oxygenase subunit OS=*Acinetobacter baumannii* (strain ATCC 17978 / CIP 53.77 / LMG 1025 / NCDC KC755 / 5377) GN=A1S_2971 PE=4 SV=2 - [A3M8Y0_ACIBT]	1.70
A3M5F8	Putative ferric siderophore receptor protein OS=*Acinetobacter baumannii* (strain ATCC 17978 / CIP 53.77 / LMG 1025 / NCDC KC755 / 5377) GN=A1S_1725 PE=3 SV=2 - [A3M5F8_ACIBT]	1.70
A3M2R7	Putative membrane protein OS=*Acinetobacter baumannii* (strain ATCC 17978 / CIP 53.77 / LMG 1025 / NCDC KC755 / 5377) GN=A1S_0771 PE=4 SV=2 - [A3M2R7_ACIBT]	1.70
A3M944	Putative membrane protein OS=*Acinetobacter baumannii* (strain ATCC 17978 / CIP 53.77 / LMG 1025 / NCDC KC755 / 5377) GN=A1S_3041 PE=4 SV=2 - [A3M944_ACIBT]	1.71
A3M3M5	Uncharacterized protein OS=*Acinetobacter baumannii* (strain ATCC 17978 / CIP 53.77 / LMG 1025 / NCDC KC755 / 5377) GN=A1S_1089 PE=3 SV=2 - [A3M3M5_ACIBT]	1.72
A3M5Y0	Uncharacterized protein OS=*Acinetobacter baumannii* (strain ATCC 17978 / CIP 53.77 / LMG 1025 / NCDC KC755 / 5377) GN=A1S_1897 PE=4 SV=2 - [A3M5Y0_ACIBT]	1.72
A3M4F1	Uncharacterized protein OS=*Acinetobacter baumannii* (strain ATCC 17978 / CIP 53.77 / LMG 1025 / NCDC KC755 / 5377) GN=A1S_1367 PE=4 SV=2 - [A3M4F1_ACIBT]	1.73

A3M9G3	Lipase OS=*Acinetobacter baumannii* (strain ATCC 17978 / CIP 53.77 / LMG 1025 / NCDC KC755 / 5377) GN=A1S_3160 PE=4 SV=2 - [A3M9G3_ACIBT]	1.74
A3M5N0	Aldehyde dehydrogenase OS=*Acinetobacter baumannii* (strain ATCC 17978 / CIP 53.77 / LMG 1025 / NCDC KC755 / 5377) GN=A1S_1797 PE=4 SV=1 - [A3M5N0_ACIBT]	1.74
A3M236	Putative holo-(Acyl carrier protein) synthase 2 OS=*Acinetobacter baumannii* (strain ATCC 17978 / CIP 53.77 / LMG 1025 / NCDC KC755 / 5377) GN=A1S_0527 PE=4 SV=2 - [A3M236_ACIBT]	1.74
A3MA44	VirP protein OS=*Acinetobacter baumannii* (strain ATCC 17978 / CIP 53.77 / LMG 1025 / NCDC KC755 / 5377) GN=A1S_3399 PE=4 SV=2 - [A3MA44_ACIBT]	1.75
A3M1N5	General secretion pathway protein F OS=*Acinetobacter baumannii* (strain ATCC 17978 / CIP 53.77 / LMG 1025 / NCDC KC755 / 5377) GN=A1S_0369 PE=3 SV=2 - [A3M1N5_ACIBT]	1.75
A7FB98	Uncharacterized protein OS=*Acinetobacter baumannii* (strain ATCC 17978 / CIP 53.77 / LMG 1025 / NCDC KC755 / 5377) GN=A1S_3661 PE=4 SV=1 - [A7FB98_ACIBT]	1.77
A3M3L5	Dichlorophenol hydroxylase OS=*Acinetobacter baumannii* (strain ATCC 17978 / CIP 53.77 / LMG 1025 / NCDC KC755 / 5377) GN=A1S_1079 PE=4 SV=2 - [A3M3L5_ACIBT]	1.77
A3M0R6	Anhydro-N-acetylmuramic acid kinase OS=*Acinetobacter baumannii* (strain ATCC 17978 / CIP 53.77 / LMG 1025 / NCDC KC755 / 5377) GN=anmK PE=3 SV=2 - [A3M0R6_ACIBT]	1.78
A7FBY9	Uncharacterized protein OS=*Acinetobacter baumannii* (strain ATCC 17978 / CIP 53.77 / LMG 1025 / NCDC KC755 / 5377) GN=A1S_3902 PE=4 SV=2 - [A7FBY9_ACIBT]	1.81
A3M6J9	Uncharacterized protein OS=*Acinetobacter baumannii* (strain ATCC 17978 / CIP 53.77 / LMG 1025 / NCDC KC755 / 5377) GN=A1S_2116 PE=4 SV=1 - [A3M6J9_ACIBT]	1.82
A3M5B9	Ribonuclease D OS=*Acinetobacter baumannii* (strain ATCC 17978 / CIP 53.77 / LMG 1025 / NCDC KC755 / 5377) GN=A1S_1686 PE=4 SV=2 - [A3M5B9_ACIBT]	1.83
A3M0Z5	Putative transcriptional regulator (LysR family) OS=*Acinetobacter baumannii* (strain ATCC 17978 / CIP 53.77 / LMG 1025 / NCDC KC755 / 5377) GN=A1S_0100 PE=4 SV=1 - [A3M0Z5_ACIBT]	1.83
A3M754	Putative nitrate transporter transmembrane protein (MFS superfamily) OS=*Acinetobacter baumannii* (strain ATCC 17978 / CIP 53.77 / LMG 1025 / NCDC KC755 / 5377) GN=A1S_2326 PE=4 SV=2 - [A3M754_ACIBT]	1.83
A3M7B7	Putative acinetobactin utilization protein OS=*Acinetobacter baumannii* (strain ATCC 17978 / CIP 53.77 / LMG 1025 / NCDC KC755 / 5377) GN=A1S_2392 PE=4 SV=2 - [A3M7B7_ACIBT]	1.84

A3M6K4	UPF0756 membrane protein A1S_2121 OS=*Acinetobacter baumannii* (strain ATCC 17978 / CIP 53.77 / LMG 1025 / NCDC KC755 / 5377) GN=A1S_2121 PE=3 SV=2 - [Y2121_ACIBT]	1.86
A3M910	Stringent starvation protein B OS=*Acinetobacter baumannii* (strain ATCC 17978 / CIP 53.77 / LMG 1025 / NCDC KC755 / 5377) GN=A1S_3003 PE=4 SV=2 - [A3M910_ACIBT]	1.86
A3M7V7	MFS family drug transporter OS=*Acinetobacter baumannii* (strain ATCC 17978 / CIP 53.77 / LMG 1025 / NCDC KC755 / 5377) GN=A1S_2584 PE=4 SV=2 - [A3M7V7_ACIBT]	1.86
A3M3A1	Putative transcriptional regulator (AraC family) OS=*Acinetobacter baumannii* (strain ATCC 17978 / CIP 53.77 / LMG 1025 / NCDC KC755 / 5377) GN=A1S_0963 PE=4 SV=2 - [A3M3A1_ACIBT]	1.86
A3M539	Uncharacterized protein OS=*Acinetobacter baumannii* (strain ATCC 17978 / CIP 53.77 / LMG 1025 / NCDC KC755 / 5377) GN=A1S_1606 PE=4 SV=1 - [A3M539_ACIBT]	1.89
A3M833	Glycerophosphoryl diester phosphodiesterase OS=*Acinetobacter baumannii* (strain ATCC 17978 / CIP 53.77 / LMG 1025 / NCDC KC755 / 5377) GN=A1S_2661 PE=4 SV=2 - [A3M833_ACIBT]	1.89
A3M7T9	Putative ferric siderophore receptor protein OS=*Acinetobacter baumannii* (strain ATCC 17978 / CIP 53.77 / LMG 1025 / NCDC KC755 / 5377) GN=A1S_2566 PE=3 SV=2 - [A3M7T9_ACIBT]	1.92
A7FBD9	Uncharacterized protein OS=*Acinetobacter baumannii* (strain ATCC 17978 / CIP 53.77 / LMG 1025 / NCDC KC755 / 5377) GN=A1S_3702 PE=4 SV=2 - [A7FBD9_ACIBT]	1.92
A3M5T4	Penicillin G amidase OS=*Acinetobacter baumannii* (strain ATCC 17978 / CIP 53.77 / LMG 1025 / NCDC KC755 / 5377) GN=A1S_1851 PE=4 SV=2 - [A3M5T4_ACIBT]	1.93
A3M9V2	Putative transcriptional regulator (Lrp-like) OS=*Acinetobacter baumannii* (strain ATCC 17978 / CIP 53.77 / LMG 1025 / NCDC KC755 / 5377) GN=A1S_3307 PE=4 SV=2 - [A3M9V2_ACIBT]	1.94
A3M4S8	Uncharacterized protein OS=*Acinetobacter baumannii* (strain ATCC 17978 / CIP 53.77 / LMG 1025 / NCDC KC755 / 5377) GN=A1S_1494 PE=4 SV=2 - [A3M4S8_ACIBT]	1.97
A3M4G2	Putative long chain fatty-acid CoA ligase OS=*Acinetobacter baumannii* (strain ATCC 17978 / CIP 53.77 / LMG 1025 / NCDC KC755 / 5377) GN=A1S_1378 PE=4 SV=2 - [A3M4G2_ACIBT]	2.02
A3M1P9	DNA gyrase inhibitor YacG OS=*Acinetobacter baumannii* (strain ATCC 17978 / CIP 53.77 / LMG 1025 / NCDC KC755 / 5377) GN=yacG PE=3 SV=1 - [A3M1P9_ACIBT]	2.02
A3M3W0	DNA polymerase V component OS=*Acinetobacter baumannii* (strain ATCC 17978 / CIP 53.77 / LMG 1025 / NCDC KC755 / 5377) GN=A1S_1174 PE=4 SV=1 - [A3M3W0_ACIBT]	2.06

A3M7R9	Secreted trypsin-like serine protease OS=*Acinetobacter baumannii* (strain ATCC 17978 / CIP 53.77 / LMG 1025 / NCDC KC755 / 5377) GN=A1S_2546 PE=4 SV=2 - [A3M7R9_ACIBT]	2.07
A3M6Y3	Uncharacterized protein OS=*Acinetobacter baumannii* (strain ATCC 17978 / CIP 53.77 / LMG 1025 / NCDC KC755 / 5377) GN=A1S_2254 PE=4 SV=2 - [A3M6Y3_ACIBT]	2.08
A3M3Y0	Uncharacterized protein OS=*Acinetobacter baumannii* (strain ATCC 17978 / CIP 53.77 / LMG 1025 / NCDC KC755 / 5377) GN=A1S_1194 PE=4 SV=2 - [A3M3Y0_ACIBT]	2.09
A3M6A5	Putative tail fiber OS=*Acinetobacter baumannii* (strain ATCC 17978 / CIP 53.77 / LMG 1025 / NCDC KC755 / 5377) GN=A1S_2022 PE=4 SV=2 - [A3M6A5_ACIBT]	2.10
A3M598	Putative membrane protein OS=*Acinetobacter baumannii* (strain ATCC 17978 / CIP 53.77 / LMG 1025 / NCDC KC755 / 5377) GN=A1S_1665 PE=3 SV=2 - [A3M598_ACIBT]	2.12
A3M2F8	Ferrous iron transport protein B OS=*Acinetobacter baumannii* (strain ATCC 17978 / CIP 53.77 / LMG 1025 / NCDC KC755 / 5377) GN=A1S_0653 PE=3 SV=1 - [A3M2F8_ACIBT]	2.16
A3M5P4	Uncharacterized protein OS=*Acinetobacter baumannii* (strain ATCC 17978 / CIP 53.77 / LMG 1025 / NCDC KC755 / 5377) GN=A1S_1811 PE=4 SV=2 - [A3M5P4_ACIBT]	2.17
A3M5L0	Methylenetetrahydrofolate reductase OS=*Acinetobacter baumannii* (strain ATCC 17978 / CIP 53.77 / LMG 1025 / NCDC KC755 / 5377) GN=A1S_1777 PE=3 SV=1 - [A3M5L0_ACIBT]	2.17
A3M1I7	Putative fusaric acid resistance protein OS=*Acinetobacter baumannii* (strain ATCC 17978 / CIP 53.77 / LMG 1025 / NCDC KC755 / 5377) GN=A1S_0317 PE=4 SV=2 - [A3M1I7_ACIBT]	2.25
A3M6H7	Uncharacterized protein OS=*Acinetobacter baumannii* (strain ATCC 17978 / CIP 53.77 / LMG 1025 / NCDC KC755 / 5377) GN=A1S_2094 PE=4 SV=2 - [A3M6H7_ACIBT]	2.28
A7FBR2	Uncharacterized protein OS=*Acinetobacter baumannii* (strain ATCC 17978 / CIP 53.77 / LMG 1025 / NCDC KC755 / 5377) GN=A1S_3825 PE=4 SV=2 - [A7FBR2_ACIBT]	2.30
A3M0Y1	Uncharacterized protein OS=*Acinetobacter baumannii* (strain ATCC 17978 / CIP 53.77 / LMG 1025 / NCDC KC755 / 5377) GN=A1S_0086 PE=4 SV=2 - [A3M0Y1_ACIBT]	2.32
A3M594	Fructose-26-bisphosphatase OS=*Acinetobacter baumannii* (strain ATCC 17978 / CIP 53.77 / LMG 1025 / NCDC KC755 / 5377) GN=A1S_1661 PE=4 SV=2 - [A3M594_ACIBT]	2.32
A3M166	NADPH-dependent FMN reductase OS=*Acinetobacter baumannii* (strain ATCC 17978 / CIP 53.77 / LMG 1025 / NCDC KC755 / 5377) GN=A1S_0179 PE=4 SV=2 - [A3M166_ACIBT]	2.37

A3M6U3	Uncharacterized protein OS=*Acinetobacter baumannii* (strain ATCC 17978 / CIP 53.77 / LMG 1025 / NCDC KC755 / 5377) GN=A1S_2210 PE=4 SV=2 - [A3M6U3_ACIBT]	2.38
A3M525	Putative Phage head-tail adaptor OS=*Acinetobacter baumannii* (strain ATCC 17978 / CIP 53.77 / LMG 1025 / NCDC KC755 / 5377) GN=A1S_1592 PE=4 SV=1 - [A3M525_ACIBT]	2.40
A3M6S8	Uncharacterized protein OS=*Acinetobacter baumannii* (strain ATCC 17978 / CIP 53.77 / LMG 1025 / NCDC KC755 / 5377) GN=A1S_2195 PE=4 SV=1 - [A3M6S8_ACIBT]	2.43
A7FBB9	Uncharacterized protein OS=*Acinetobacter baumannii* (strain ATCC 17978 / CIP 53.77 / LMG 1025 / NCDC KC755 / 5377) GN=A1S_3682 PE=4 SV=1 - [A7FBB9_ACIBT]	2.44
A3M5Q5	Putative transcription regulator protein OS=*Acinetobacter baumannii* (strain ATCC 17978 / CIP 53.77 / LMG 1025 / NCDC KC755 / 5377) GN=A1S_1822 PE=4 SV=2 - [A3M5Q5_ACIBT]	2.48
A3MA53	Uncharacterized protein OS=*Acinetobacter baumannii* (strain ATCC 17978 / CIP 53.77 / LMG 1025 / NCDC KC755 / 5377) GN=A1S_3408 PE=4 SV=2 - [A3MA53_ACIBT]	2.62
A3M7D5	Fatty acid desaturase OS=*Acinetobacter baumannii* (strain ATCC 17978 / CIP 53.77 / LMG 1025 / NCDC KC755 / 5377) GN=A1S_2410 PE=4 SV=2 - [A3M7D5_ACIBT]	2.75
A7FBC0	Uncharacterized protein OS=*Acinetobacter baumannii* (strain ATCC 17978 / CIP 53.77 / LMG 1025 / NCDC KC755 / 5377) GN=A1S_3683 PE=4 SV=2 - [A7FBC0_ACIBT]	2.88
A7FAV6	Uncharacterized protein OS=*Acinetobacter baumannii* (strain ATCC 17978 / CIP 53.77 / LMG 1025 / NCDC KC755 / 5377) GN=A1S_3519 PE=4 SV=2 - [A7FAV6_ACIBT]	2.95
A3M1D5	Permease (DMT) superfamily OS=*Acinetobacter baumannii* (strain ATCC 17978 / CIP 53.77 / LMG 1025 / NCDC KC755 / 5377) GN=A1S_0254 PE=4 SV=2 - [A3M1D5_ACIBT]	3.01
A3M8U0	Putative cation efflux system protein OS=*Acinetobacter baumannii* (strain ATCC 17978 / CIP 53.77 / LMG 1025 / NCDC KC755 / 5377) GN=A1S_2929 PE=4 SV=2 - [A3M8U0_ACIBT]	3.70
A3M4M1	Putative acyl-CoA dehydrogenase OS=*Acinetobacter baumannii* (strain ATCC 17978 / CIP 53.77 / LMG 1025 / NCDC KC755 / 5377) GN=A1S_1437 PE=4 SV=1 - [A3M4M1_ACIBT]	4.30
A3M8N5	Uncharacterized protein OS=*Acinetobacter baumannii* (strain ATCC 17978 / CIP 53.77 / LMG 1025 / NCDC KC755 / 5377) GN=A1S_2873 PE=4 SV=1 - [A3M8N5_ACIBT]	5.01
A3M2Q8	NADH dehydrogenase I chain L OS=*Acinetobacter baumannii* (strain ATCC 17978 / CIP 53.77 / LMG 1025 / NCDC KC755 / 5377) GN=A1S_0762 PE=4 SV=2 - [A3M2Q8_ACIBT]	5.37

Table S2. Transcription factor binding boxes in *ompW* promotor.

Transcription factor	Consensus box	Position
argR	AATTAATA	10
phoB	AATAAAAG	14
rpoD17	TAGCCTTT	33
lrp	TTTCTTTT	39
ompR	TTCTTTTT	40
argR	TTTTTTAT	43
argR2	TTTTTATT	44
ihf	TTTTATTT	45
argR2	TTTATTTT	46
rpoD17	ATTTTGTA	49
argR2	TTTTTATT	58
phoB	TTTATTAA	60
arcA	AATAAAAA	66
argR	ATAAAAAT	67

Table S3. Primers used in this study.

Primer name	Sequence
OmpW-pMJG42-SpeI-Up-F	GGGCCCACTAGTGACAAGCCTGCGTCAAAACGC
OmpW-pMJG42-BamHI-Up-R	GGGCCCGGATCCTTTCATCATAAAAGGACTCCATGTCCG
OmpW-pMJG42-BamHI-Down-F	GGGCCCGGATCCCGATTCTAAATCTCAAAAGCACAC
OmpW-pMJG42-NotI-Down-R	GGGCCCGCGGCCGCGTCGTACACCTAGTGACAAAGCC
OmpW-Out-F	GGTGACAAATGGACAGTTGTAAC
OmpW-Out-R	AGCGGACATCTTTGGCCGTC
NotI-OmpW-F	ACAGCGGCCGCTTTAATCAAAATTAATAAAAGGCTGC
XbaI-OmpW-R	ACATCTAGAACGCCGTCCCCCTGAACTGA
Seq-insert-pUCp24-F	TCCCAGTCACGACGTTGTAAAACG
Seq-insert-pUCp24-R	AATTTCACACAGGAAACAGCTATG
